# A presentation of spinal epidural abscess in a seven-year-old VACTERL patient: a case report

**DOI:** 10.1186/s13256-026-06356-6

**Published:** 2026-07-19

**Authors:** Sarah Kebbeh, Sana Masoud, Mary Alakkash, Manar Abdullah

**Affiliations:** 1 Faculty of Medicine, University of Aleppo, Aleppo, Syria; 2 Pediatrics Department, Faculty of Medicine, University of Aleppo, Aleppo, Syria

**Keywords:** Spinal epidural abscess, VACTERL association, Patent ductus arteriosus (PDA), Case report, Scoliosis, Umbilical hernia

## Abstract

**Background:**

Spinal epidural abscess is a rare but potentially life-threatening infection of the central nervous system. VACTERL association is a rare congenital condition characterized by vertebral, anal, cardiac, tracheoesophageal, renal, and limb anomalies.

**Case presentation:**

We report a 7-year-old Arab male with VACTERL association presenting with back pain, fever, and gait difficulty. Laboratory studies showed leukocytosis (27 × 10⁹/L) and anemia (Hb 9 g/dL). MRI revealed multiloculated cystic lesions with thick walls and mixed fluid–gas content extending from T4 to L5, forming an extensive epidural abscess with paraspinal extension and vertebral erosions. The patient was treated with intravenous vancomycin, metronidazole, and imipenem, followed by surgical drainage and local vancomycin irrigation. Clinical condition improved markedly, and he was discharged after two months in good health.

**Conclusion:**

This case highlights the diagnostic challenges of SEA in children with complex congenital anomalies and emphasizes the importance of early recognition and multidisciplinary management.

## Introduction

An epidural abscess is uncommon but can be a life-threatening condition that requires prompt diagnosis and immediate management. An epidural abscess is an inflammation that contains an accumulation of pus between the dura and the skull or spine bones. There are two types of epidural abscess, spinal epidural abscess (SEA) and intracranial epidural abscess (IEA) [1]. The difference comprises where it forms in the CNS, and some variants in risk factors and symptoms [1]. The estimated incidence of epidural abscess occurring in large tertiary care centers is approximately 0.2–2.8 cases per 10,000 admissions [1].

VACTERL association, also called Vater syndrome, involves several conditions that appear together: the vertebrae, anus, cardiac anomalies, trachea, esophagus, renal anomalies, and limb differences [2]. The name is based on an abbreviation for the organs and systems that are affected. The clinical presentation of VACTERL association occurs varies among affected individuals. The combination of symptoms or the severity of symptoms experienced by patients are different. It is estimated that 1 out of 10,000 to 40,000 babies is born with VACTERL [2]. Because the condition presents in different ways and diagnostic criteria vary, it is likely the condition is somewhat underdiagnosed [2]. In this report, we describe the coexistence of spinal epidural abscess and VACTERL association in a single patient.

## Case presentation

A 7-year-old Arab male child presented to our practice with complaints of back pain radiating to the shoulders, lumbar region, and hips, accompanied by continuous fever, generalized fatigue, and difficulty walking over the past two weeks.

### History

The antenatal period was unremarkable. However, after birth, physical examination revealed “Anal atresia” and “Umbilical Hernia.” At the age of 3 months, the child was diagnosed with patent ductus arteriosus (PDA) for which surgical correction was indicated. At 1.5 years of age, a left nephrectomy was performed due to a teratoma. The patient also exhibited delayed psychomotor development.

### Family history

There was a third-degree paternal consanguinity, and the patient’s uncle had a history of anal atresia.

### Physical examination

**Fig.1:**
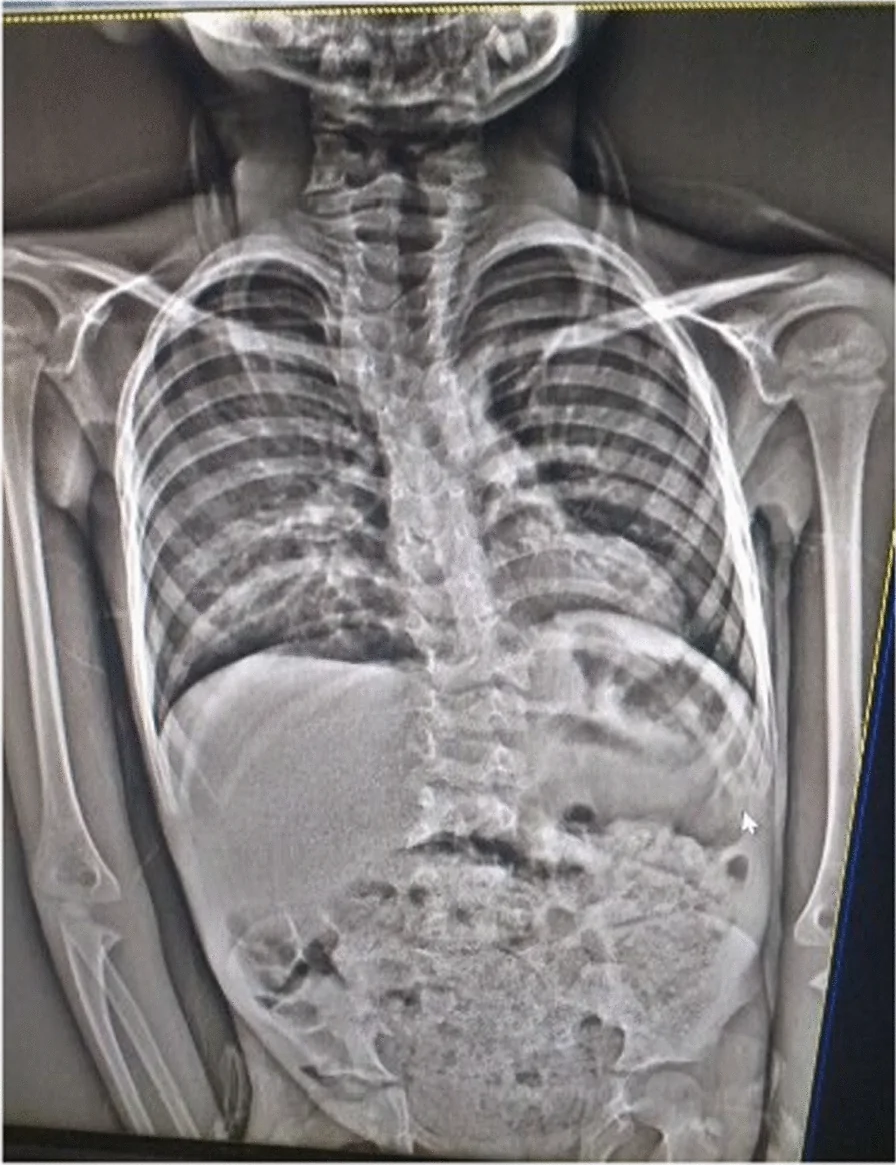
The X-ray image showed a scoliosis with the primary curve toward the right and its apex at T12, and the compensatory curve toward the left with its apex at L4 and L5

Cardiopulmonary auscultation was normal. The child had scoliosis (Fig. 1).

Anthropometric measurements showed a weight of 16.5 kg, height of 108 cm, and head circumference of 49 cm. According to WHO growth charts, the child’s weight, height, and head circumference were all below the 3rd percentile, indicating growth failure.

Neurological examination revealed mild gait instability but preserved muscle strength and sensation in all limbs. No sphincter dysfunction was noted.

### Laboratory findings

Urea: 18 mg/dL

Creatinine: 0.3 mg/dL

Sodium (Na⁺): 130 mmol/L

Potassium (K⁺): 4.1 mmol/L

Calcium (Ca2⁺): 1.11 mmol/L

Hemoglobin: 9 g/dL → increased to 10.1 g/dL after transfusion of 200 mL whole blood.

### Peripheral blood smear

RBCs: Erythrocytopenia, anisocytosis, some microcytic and slightly to moderately hypochromic cells, poikilocytosis (target cells, stomatocytes)

WBCs: Leukocytosis with neutrophilia and left shift (presence of many band forms and metamyelocytes)

Platelets: Normal morphology

### CBC values

WBC: 27.17 × 10⁹/L → decreased to 17.1 × 10⁹/L after treatment

PLT: 433 × 10⁹/L

MID#: 1.98

LYM#: 2.61

GRAN#: 22.58

MID%: 7.3

LYM%: 9.6

GRAN%: 83.1

### Culture result

Pus culture did not yield any bacterial growth.

### Imaging

Abdominal ultrasound revealed multiple fluid collections with thickened walls, suggesting abscess formation adjacent to the spinal column.

Spinal magnetic resonance imaging (MRI) demonstrated multiple multiloculated cystic lesions with thick, irregular walls extending along both thoracic and lumbar regions. The largest lesion was located on the right paraspinal area, measuring approximately 6.5 cm in diameter, showing mixed fluid–gas levels. These lesions exhibited intermediate signal intensity on T1-weighted, high signal on T2-weighted sequences, restricted diffusion, and marked ring enhancement after contrast injection.

The abscesses communicated with the posterior epidural space, forming an extensive epidural abscess extending from T4 to L5, with a maximal thickness of 8 mm and mild compression of the dural sac.

There was also extension into the right psoas and paraspinal muscles with a small subphrenic collection. The MRI revealed vertebral body erosions at T7, T10–T11, and L4–L5, partial vertebral fusion, and right-convex thoracic scoliosis centered at T12 with compensatory lumbar curvature.

**Fig.2:**
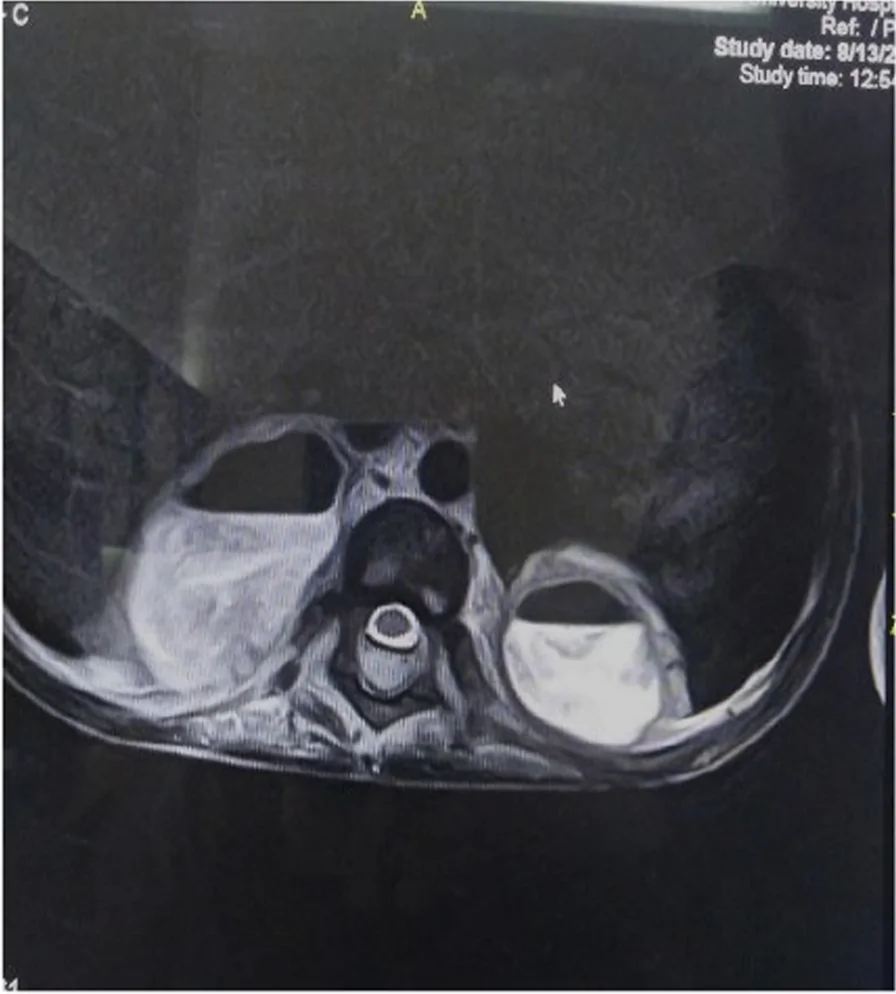
Multiple cystic formations were observed on the sides of the thoracic and lumbar spine, which exhibited significant circumferential enhancement after injection

**Fig.3:**
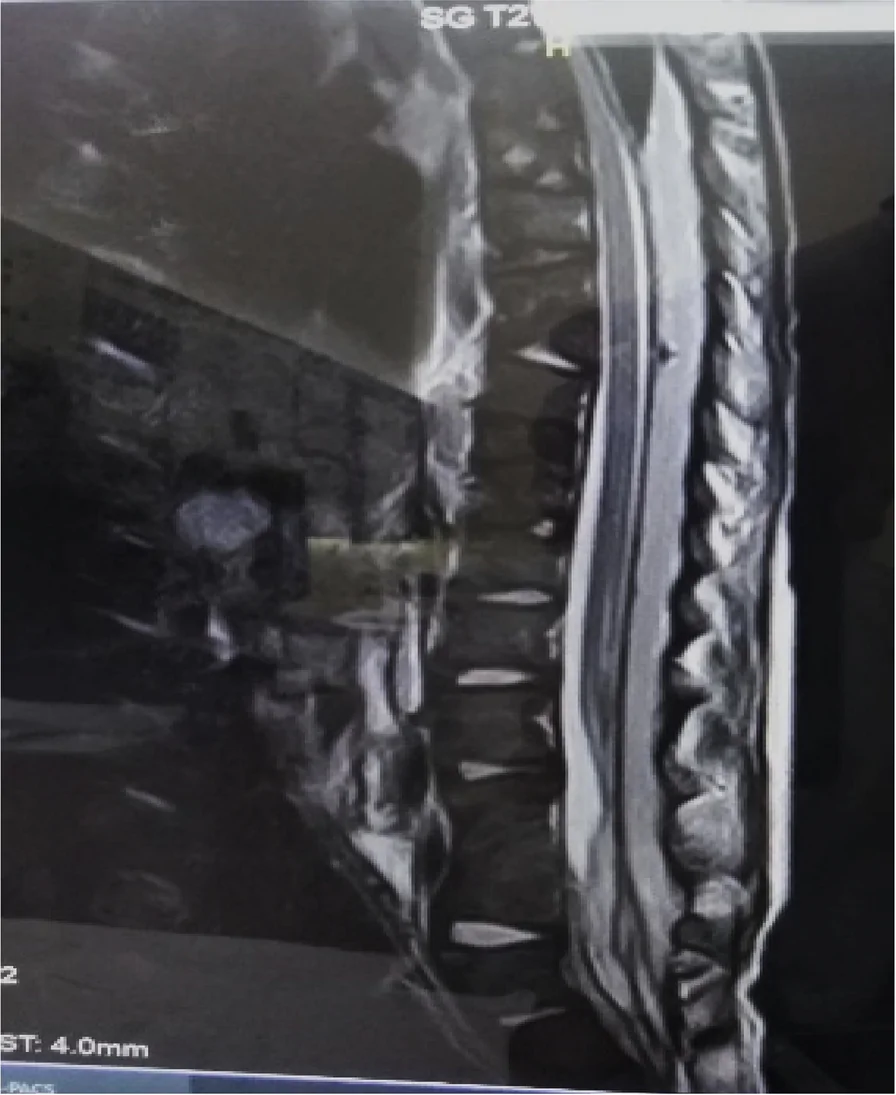
Epidural abscess Located within the spinal canal out of the dural sac, extending along the entire spinal canal, with the presence of a fluid-filled cavity in its upper portion

The overall findings were consistent with infectious spondylodiscitis complicated by an extensive epidural abscess in the setting of congenital spinal malformations (Figs. 2 and 3).

### Treatment

Initial management included intravenous antibiotic therapy with vancomycin, metronidazole (Flagyl), and imipenem, in addition to intravenous fluids at half maintenance rate.

After clinical improvement, therapy was adjusted to vancomycin, metronidazole, and ciprofloxacin, and the patient tolerated an adequate oral intake.

Body temperature improved from 39.5 °C to 36.6 °C following treatment.

### Surgical report

Under general anesthesia and in the prone position, after standard triple povidone-iodine sterilization and sterile draping, a midline skin incision was made over the spinous processes from T4 to L5. Muscular dissection was performed to expose the spinous processes and laminae of the affected vertebrae.

Laminectomy was carried out at the levels of T5, T9, T12, and L3, resulting in the evacuation of purulent material from the epidural space. The abscess cavity was thoroughly irrigated with normal saline and hydrogen peroxide, and local hemostasis was achieved. The wound was then irrigated with vancomycin 1 g solution, followed by placement of two drainage tubes (black suction drains). The wound was closed in layers and covered with a sterile dressing.

### Outcome

The postoperative course was favorable, with marked clinical improvement. The patient’s WBC count decreased, fever resolved, and overall condition improved gradually. The child was discharged after two months of hospitalization in good general condition (Table 1 summarizes clinical timeline).

**Table 1:** Timeline of clinical events for the patient

Time point	Clinical event
T0	Onset of back pain, persistent fever, and gait difficulty
T0 + 2 days	Hospital admission and initial laboratory investigations
T0 + 1 weeks	Spinal MRI revealed extensive epidural abscess from T4 to L5
T0 + 3 days	Initiation of broad-spectrum intravenous antibiotics
T0 + 2 weeks	Surgical drainage and laminectomy performed
T0 + 2 months	Clinical improvement and discharge in good general condition

Clinical events are presented using relative time point (T0) to maintain patient anonymity

The diagnosis in this case was particularly challenging due to the nonspecific initial presentation and the patient’s complex congenital background. Overlapping symptoms related to pre-existing vertebral anomalies and growth failure may obscure early signs of infection. Furthermore, limited access to advanced imaging modalities can contribute to delayed diagnosis in pediatric patients. This case emphasizes the importance of maintaining a high index of suspicion for spinal epidural abscess in children with congenital syndromes who present with persistent back pain and fever.

## Discussion and conclusions

Spinal epidural abscess (SEA) in children is extremely rare and may present with nonspecific symptoms, making early diagnosis challenging [3]. The coexistence of SEA with congenital syndromes such as VACTERL association has not been reported previously to our knowledge [4]. This case highlights the importance of maintaining a high index of suspicion for SEA in children with complex congenital anomalies presenting with back pain and fever.

The pathophysiology of spinal epidural abscess involves bacterial seeding of the epidural space through hematogenous spread, direct extension from adjacent structures, or iatrogenic introduction.

In pediatric patients, immature immune responses and previous systemic infections may increase susceptibility to such infections.

Furthermore, vertebral anomalies and scoliosis, commonly observed in VACTERL association, may alter spinal anatomy and local vascular supply, potentially facilitating microbial localization and impairing immune clearance.

Recurrent hospitalizations and prior episodes of bacteremia may further contribute to colonization and subsequent abscess formation in susceptible individuals.

Risk factors are present in less than a third of pediatric cases: sickle cell anemia, leukemia, long-term use of steroids, and immunosuppression [5]. In our case, there was no obvious risk factor. We did not find any study involves spinal epidural abscess with scoliosis, other vertebral anomalies, or even with VACTERL association. Meningitis and bacteremia at the age of 3 years may have a role in this case. However, all tests, imaging, and physical exams suggest a spontaneous spinal epidural abscess.

However, several reports have also described spontaneous cases without identifiable risk factors [7].

Spinal epidural abscess is considered a rare disease, with an estimated incidence of 0.2–2.8 cases per 10,000 admissions to large tertiary care centers. It is most common in males and has higher prevalence in the fifth to seventh decade of life. In our case, the patient was only seven years old, which is infrequently reported [6]. Remarkably, the co-occurrence of the spinal epidural abscess with the VACTERL syndrome, which is also a rare disease of an estimated incidence of 1 in 100,000–40000 of newborns [2], will be a new insight into understanding the pathophysiology of spinal epidural abscess on a lining scoliosis as a symptom of VACTERL syndrome.

Large case series have emphasized that delayed diagnosis is common due to nonspecific clinical manifestations, which may lead to increased neurological morbidity [7].

Compared with previously published pediatric cases, our patient presented at a younger age and lacked conventional risk factors, making this case particularly unusual.

Moreover, the coexistence of SEA with VACTERL association has not been documented in available literature, further emphasizing the uniqueness of this report.

Studies revealed evidence for an inherited component in a subset of patients, they observed that 10% of VACTERL patient’s first-degree relatives have VACTERL association [7]. However, in our case, the uncle (second degree relative) had only an anal atresia.

VACTERL association is believed to result from disturbances in mesodermal development during early embryogenesis, possibly involving disruption of molecular signaling pathways such as Sonic Hedgehog.

These developmental abnormalities lead to structural defects of the vertebral column and surrounding tissues, which may predispose affected individuals to chronic mechanical stress and microvascular compromise.

Such alterations could theoretically facilitate bacterial adherence and invasion of epidural tissues, although this relationship remains speculative and requires further investigation [8].

This case underscores the importance of considering spinal epidural abscess in children with complex congenital anomalies who present with nonspecific symptoms.

Early utilization of magnetic resonance imaging is essential for timely diagnosis and appropriate management.

Prompt initiation of antimicrobial therapy combined with surgical intervention, when indicated, remains the cornerstone of successful treatment.

In conclusion, spinal epidural abscess in children is a rare but potentially devastating condition.

Our report describes a previously unreported coexistence of SEA and VACTERL association and emphasizes the need for early recognition, comprehensive evaluation, and multidisciplinary management.

Further multicenter studies and additional case reports are required to elucidate the underlying mechanisms linking congenital vertebral anomalies and infectious processes.

## Data Availability

Not applicable.

## Abbreviations

CNS: Central nervous system
PDA: Patent ductus arteriosus
SEA: Spinal epidural abscess
IEA: Intracranial epidural abscess

## References

[1] Mark R Wallace, MD, FACP, FIDSA Infectious Disease Physician, Skagit Valley Hospital SRH. Epidural Abscess. medscape. Published 2022. https://emedicine.medscape.com/article/232570-overview#a4

[2] Suzi and Scott Lustgarten Center for GI Motility. VACTERL Association (VATER syndrome). https://www.chop.edu/conditions-diseases/vacterl-association-vater-syndrome.

[3] Danner RL, Hartman BJ. Update on spinal epidural abscess: 35 years of experience. N Engl J Med. 2002;347(12):913–20.

[4] Sendi P, Bregenzer T, Zimmerli W. Spinal epidural abscess in clinical practice. QJM. 2008;101(1):1–12. 10.1093/qjmed/hcm100 17982180

[5] Fox sean M. spinal epidural abscess in children. pedemmorsels. Published 2020. https://pedemmorsels.com/spinal-epidural-abscess-in-children/

[6] Omaditya (Goldey) Khanna, MD Resident Physician, Department of Neurological Surgery TJUH. Spinal Epidural Abscess: Background, Pathophysiology, Epidemiology. medscape. Published 2022. https://emedicine.medscape.com/article/1165840-overview#a5

[7] Khursheed N, Dar S, Ramzan A, Fomda B, Humam N, Abrar W, *et al*. Spinal epidural abscess: report on 27 cases. Surg Neurol Int. 2017;8(1):240. 10.1186/1750-1172-6-56. PMC5629861 29026676

[8] Solomon BD. The etiology of VACTERL association: Current knowledge and hypotheses. Vol. 178, American Journal of Medical Genetics, Part C: Seminars in Medical Genetics. Blackwell Publishing Inc.; 2018. p. 440–6. 10.1002/ajmg.c.31664 30580478

